# Technical success, resection status, and procedural complication rate of colonoscopic full-wall resection: a pooled analysis from 7 hospitals of different care levels

**DOI:** 10.1007/s00464-020-07772-5

**Published:** 2020-07-09

**Authors:** Irmengard Krutzenbichler, Markus Dollhopf, Helmut Diepolder, Andreas Eigler, Martin Fuchs, Simon Herrmann, Gerhard Kleber, Björn Lewerenz, Christoph Kaiser, Tilman Lilje, Timo Rath, Ayman Agha, Francesco Vitali, Claus Schäfer, Wolfgang Schepp, Felix Gundling

**Affiliations:** 1 Department of Gastroenterology, Hepatology and Gastrointestinal Oncology, Bogenhausen Academic Teaching Hospital, Technical University of Munich, Munich, Germany; 2 Division of Gastroenterology and Hepatology, Klinikum Neuperlach, Munich, Germany; 3 Kliniken Ostallgäu-Kaufbeuren, Klinikum Kaufbeuren, Germany; 4 Klinik für Innere Medizin I, Klinikum Dritter Orden München-Nymphenburg, Munich, Germany; 5 Medical Department I, Kliniken Ostalb, Aalen, Germany; 6 Ludwig Demling Endoscopy Center of Excellence, Division of Gastroenterology, Friedrich-Alexander-University, 91054 Erlangen, Germany; 7 Klinik für Allgemein-, Viszeral-, Endokrine Und Minimal-Invasive Chirurgie, Klinikum Bogenhausen, Technical University of Munich, Munich, Germany; 8 Medical Clinic II, Klinikum Neumarkt, Neumarkt, Germany; 9 Department for Gastroenterology, Diabetics and Endocrinology, Kemperhof Hospital, Gemeinschaftsklinikum Mittelrhein, Koblenz, Germany

**Keywords:** Endoscopic full-thickness resection (eFTR), Endoscopic resection, Full-thickness resection device (FTRD®), Polypectomy, ‘WALL RESECT’

## Abstract

**Introduction:**

Endoscopic full-thickness resection (eFTR) using the full-thickness resection device (FTRD®) is a novel minimally invasive procedure that allows the resection of various lesions in the gastrointestinal tract including the colorectum. Real-world data outside of published studies are limited. The aim of this study was a detailed analysis of the outcomes of colonoscopic eFTR in different hospitals from different care levels in correlation with the number of endoscopists performing eFTR.

**Material and methods:**

In this case series, the data of all patients who underwent eFTR between November 2014 and June 2019 (performed by a total of 22 endoscopists) in 7 hospitals were analyzed retrospectively regarding rates of technical success, R0 resection, and procedure-related complications.

**Results:**

Colonoscopic eFTR was performed in 229 patients (64.6% men; average age 69.3 ± 10.3 years) mainly on the basis of the following indication: 69.9% difficult adenomas, 21.0% gastrointestinal adenocarcinomas, and 7.9% subepithelial tumors. The average size of the lesions was 16.3 mm. Technical success rate of eFTR was achieved in 83.8% (binominal confidence interval 78.4–88.4%). Overall, histologically complete resection (R0) was achieved in 77.2% (CI 69.8–83.6%) while histologically proven full-wall excidate was confirmed in 90.0% (CI 85.1–93.7%). Of the resectates obtained (*n* = 210), 190 were resected en bloc (90.5%). We did not observe a clear improvement of technical success and R0 resection rate over time by the performing endoscopists. Altogether, procedure-related complications were observed in 17.5% (mostly moderate) including 2 cases of acute gangrenous appendicitis requiring operation.

**Discussion:**

In this pooled analysis, eFTR represents a feasible, effective, and safe minimally invasive endoscopic technique.

**Electronic supplementary material:**

The online version of this article (10.1007/s00464-020-07772-5) contains supplementary material, which is available to authorized users.

Conventional endoscopic resection techniques of polyps in the colorectum such as endoscopic mucosal resection and endoscopic submucosal dissection are highly effective [1, 2]. However, they are limited to the mucosa and submucosa [1–4]. Therefore, endoscopic resection of difficult lesions (e.g., non-lifting polyps), early carcinomas, and subepithelial tumors is often difficult and sometimes not possible [4, 5]. Endoscopic full thickness resection using the full-thickness resection device (FTRD®) is a novel minimally invasive procedure allowing the resection of various lesions that were previously not conventionally resectable [6–9]. Clip-assisted endoscopic full-thickness resection (eFTR) using a special device for creating and resecting a duplicated intestinal wall has been shown to be feasible mainly for lesions in the colorectum in several small retrospective studies [10–13]. This novel and relatively simple endoscopic technique allows effective treatment of, e.g., difficult colorectal lesions and may become an alternative to surgery in selected patients [14]. To date, eFTR has been successfully performed in different regions of the gastrointestinal tract apart from the colorectum such as stomach and small intestine [15–19].

Recently, a first large prospective multicenter study (‘WALL RESECT’) demonstrated that eFTR using FTRD® system is effective for difficult-to-resect colorectal lesions, especially for non-lifting polyps and for lesions ≤ 20 mm [20]. This prospective, investigator-initiated, non-randomized clinical trial was conducted at nine referral centers in Germany [20]. In all cases, colonoscopic FTRD® was performed by skilled endoscopists with broad experience in innovative and experimental endoscopy which may explain the high total technical success rate of 89.5% while histological R0 resection could be achieved in 76.9% [20]. In this study, the complication rate was acceptable with a low rate of emergency surgery (in 2.2%, 20).

However, real-world data of eFTR performed by endoscopists in hospitals with a lower level of care (e.g., basic and standard care, main care providers) and outside of published studies are rare [21–29]. In particular, it is not clear whether the positive results of the published studies obtained from referral centers can be transferred to the performance quality in other hospitals. Therefore, the aim of the present non-investigator-initiated study was a retrospective analysis of colonoscopic FTRD®-procedures performed in 7 different hospitals in Southern Germany (secondary and tertiary care level) regarding technical success rate, en bloc (R0) resection status, and procedural complications.

## Material and methods

### Study population

There are differences between hospitals in Germany regarding their care levels [30]. The clinics participating in the present pooled analysis belonged either to secondary care level (supra-local priority tasks in diagnostics and therapy: Klinika Kaufbeuren, Neumarkt, Ostalb, Dritter Orden) or to tertiary care level (comprehensive and differentiated medical-technical facilities or university hospitals: Klinika Bogenhausen, Neuperlach, University Hospital Erlangen).

Study data were collected and analyzed at coordinating study center at Klinikum Bogenhausen. The database was created by using Microsoft Excel. Data entry and obtained by a trained student (IK) at the Department of Gastroenterology, Gastrointestinal Oncology, and Hepatology at Klinikum Bogenhausen. The source data of the participating hospitals were reviewed by the coordinating physician (FG) after completion of patient enrolment. Since data analysis was performed retrospectively, no systematic monitoring of the endoscopy database (e.g., for selection bias) was available. Therefore, inclusion of patients in the present analysis was depended on the participating hospitals.

Retrospectively, these data were recruited from hospitalized patients who underwent consecutively eFTR in the colorectum using FTRD® system (on the basis of OPS codes 5-452.25, 5-482.82, 5-452.65) during a 6-year period (from November 2014 until June 2019) from databases in the departments of gastroenterology and endoscopy of the participating hospitals.

The clinical characteristics of all patients (*n* = 229, 148 men and 81 women) are presented in Table 1. EFTR using FTRD® system was performed in patients with colorectal lesions which were difficult or not possible to resect with conventional endoscopic methods (e.g., polypectomy, EMR or ESD). Those included colorectal adenoma with negative lifting sign (recurrent, incompletely resected, or treatment naive), adenoma involving the appendiceal orifice or a diverticulum, T1 adenocarcinoma with indication for endoscopic resection, neuroendocrine tumor (NET), dysplasia associated lesion or mass (DALM), subepithelial colorectal tumor, and full-wall biopsy for diagnosis of suspected aganglionosis (Hirschsprung’s disease, Table 1).

**Table 1:** Characteristics of patient population. Comorbidities could be assessed in 159 (body mass index in 126) patients (*n* = 229 patients, *n*/%) and target lesions

Sex, *n* (%)	
Male	148 (64.6%)
Female	81 (35.4%)
Age, median (range)	69.29 (34–91)
Comorbidity, *n* (%)	
Obesity (*n* = 126)	30 (23.8%)
Diabetes mellitus (*n* = 159)	26 (16.4%)
Hypertension (*n* = 159)	64 (40.3%)
Coronary heart disease (*n* = 159)	14 (8.8%)
Stroke (*n* = 159)	2 (1.3%)
Inflammatory bowel disease (*n* = 159)	7 (4.4%)
Colonic diverticulosis (*n* = 159)	46 (28.9%)
Indication for EFTR, *n* (%)	
Adenoma with negative lifting sign	160 (69.9%)
Recurrent	53 (23.1%)
Incompletely resected	64 (27.9%)
Treatment naive	43 (18.8%)
DALM	2 (0.9%)
Highly suspected or confirmed carcinoma	48 (21.0%)
Subepithelial mass/ confirmed NET	18 (7.9%)
Biopsy for Aganglionosis (Hirschsprung’s disease)	1 (0.4%)
Location of lesion, *n* (%)	
Coecum	19 (8.3%)
Appendiceal orifice	9 (3.9%)
Ascending colon	59 (25.8%)
Transverse colon	27 (11.8%)
Descending colon	19 (8.3%)
Sigmoid	34 (14.8%)
Rectum	58 (25.3%)
Surgical anastomosis	4 (1.7%)
Other lesion characteristics	
Lesion involving a diverticulum	3 (1.3%)
Maximum diameter of lesion, mean (range, mm)	16.32 (3–50)

*DALM* dysplasia associated lesion or mass, *NET* neuroendocrine tumor

Aims of this study were the evaluation of:

- Technical success: this was defined by (a) successful advancement of the endoscope with the cap mounted to the target lesion and (b) macroscopically complete resection (no macroscopic evidence of residual lesion, confirmed by the endoscopist)
- En bloc (R0) resection rate and histologically complete resection, defined as tumor-free lateral and deep resection margins, confirmed by the pathologist
- Histologically confirmed full-thickness resection (visibility of all layers of the colonic wall including *Lamina muscularis p*ropria or *serosa* within the resection specimen). In case of carcinomas, curative resection was defined as lateral and deep R0 resection, confirmed by the pathologist
- Rate of procedure-related immediate (0–12 h) or late (12–14 days) complications after eFTR
- A possible learning curve demonstrating an improved rate of technical success and R0 resection over time, due to greater experience of the endoscopist. Therefore, the participating hospitals were analyzed regarding their volume of FTRD-endoscopies and their number of endoscopists performing eFTR

### Full-thickness resection device

As described by Schmidt et al., the FTRD® consists of an over-the-scope system which can be mounted over standard colonoscopes [6–10, 20]. It contains a transparent cap with a modified 14 mm over-the-scope clip (OTSC). Compared with a conventional OTSC system, the cap is longer (23 mm measured from the tip of the endoscope) and clip design is slightly modified [20]. The tip of the cap harbors a 13 mm snare. The handle of the snare runs on the outer surface of the endoscope underneath a transparent plastic sheath. Intestinal wall defect is closed using the integrated OTSC before resection [20]. The device has gained CE mark for eFTR in the lower GI tract in September 2014.

### EFTR procedure and periprocedural management

**Fig. 1:**
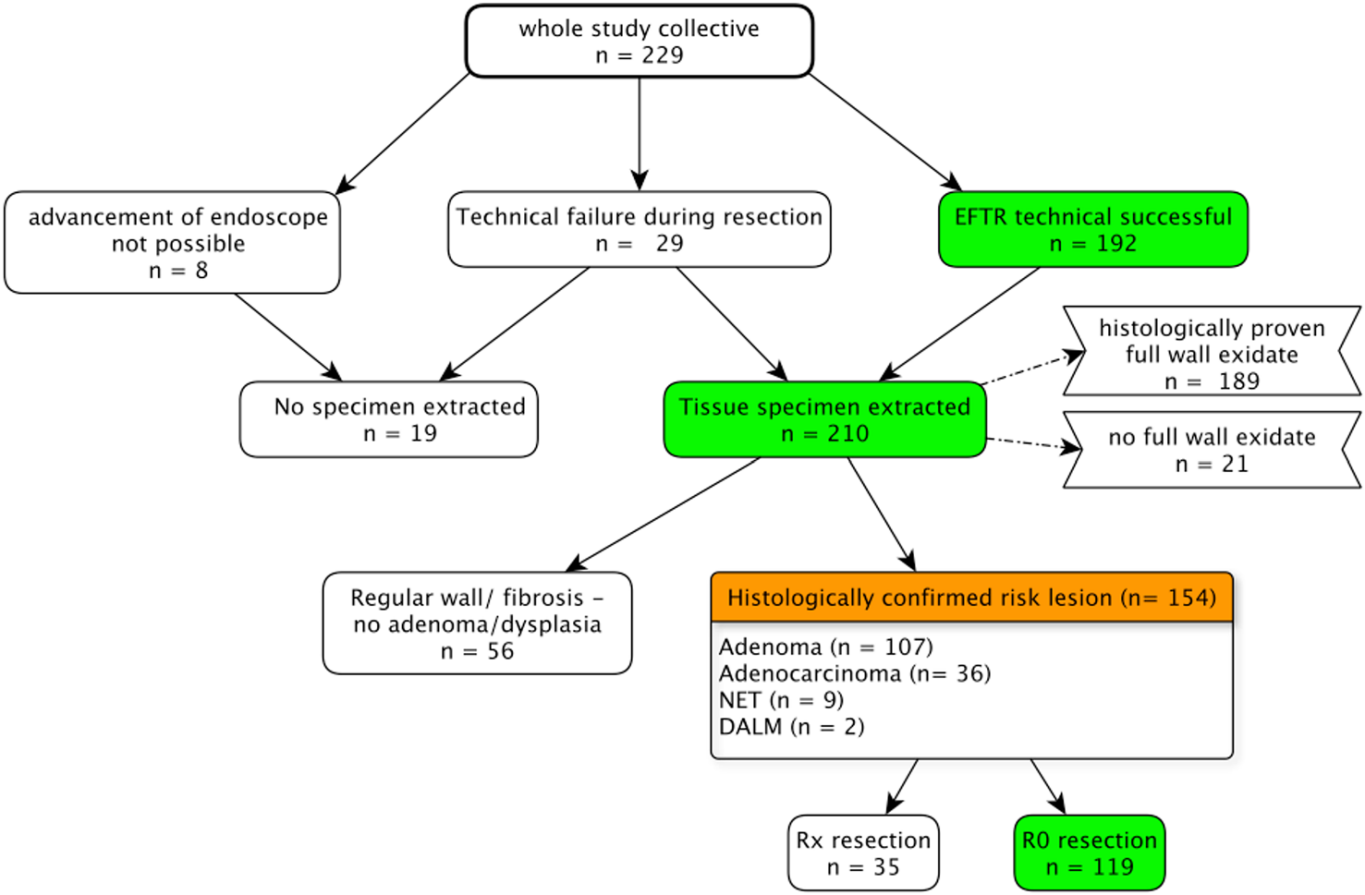
Overview of results of the whole study collective is shown as a flow chart. Lesions that required close follow-up or surgery were qualified as "risk lesions"

Endoscopic intervention of eFTR and handling of the resection specimen were performed as described by Schmidt et al. (20, Fig. 1A–D). All patients provided informed consent to undergo endoscopic resection including the full-thickness resection procedure. All procedures were done in an inpatient setting under deep sedation with propofol + / − midazolam. All patients received prophylactic antibiotic therapy starting immediately before the procedure. Blood pressure, heart rate, and oxygen saturation were constantly monitored during the procedure. *Patients in all study centers* on acetylsalicylic acid were advised to continue the medication while all other anticoagulants (e.g., clopidogrel or direct oral anticoagulants) were discontinued.

All resections were performed by endoscopists with broad expertise in endoscopy (senior physician, chief physician of the department), EMR, and OTSC placement. Most (but not all) of the participating endoscopists additionally had undergone a 1-day training in eFTR with FTRD® (as recommended by the manufacturer), which included hands-on training on ex vivo pig models before the start of this analysis.

The size of the lesions was measured endoscopically (e.g., using standardized forceps or snares with defined sizes). Lateral margins of the lesion were mostly circumferentially marked with coagulation using a high-frequency probe (Ovesco Endoscopy). Then, the endoscope with the mounted FTRD® was again advanced to the lesion. A grasping forceps or an anchor device (Ovesco Endoscopy) was advanced through the working channel. The lesion was then slowly pulled into the cap until lateral markings were visible in the cap. The clip was then deployed, and tissue above the clip was immediately resected with the snare. The resection specimen was subsequently removed, the resection site was endoscopically inspected for resection completeness and signs of complications (e.g., perforation or bleeding). The resection specimen was immerged in formalin and evaluated histologically by the local pathologist in each hospital. As recommended by OVESCO, patients were put on clear liquids at the same day if there was no clinical evidence of peritonitis and received regular diet the next day.

The study protocol was examined, approved, and accompanied not by Institutional Review Board (IRB) but by the regional Ethics Committees in the geographic area (Bayerische Ärztekammer, EK-No 2018-129 and the Universität of Ulm, No 455/18). All authors had access to the study data and reviewed and approved the final manuscript.

### Statistical analysis

Statistical analysis was performed descriptively using statistical software IBM® SPSS Statistics version 26 (2019; https://www.ibm.com/de-de/products/spss-statistics). Qualitative data are presented as absolute and relative frequencies and quantitative data as mean, median, minimum, and maximum. Furthermore, the binomial 95-percent confidence interval was calculated regarding technical success, R0 resection rate, and histologically confirmed full-thickness resection.

## Results

### Patient’s and lesional characteristics

Between November 2014 and June 2019, eFTR had been performed (by a total of 22 endoscopists) in 229 patients at 7 hospitals in Southern Germany. Patient and lesion characteristics are shown in Table 1. 148 (64.6%) of the patients were male, 81 (35.4%) were female. The average age at intervention was 69.3 ± 10.3 years. In the majority of patients (70%), comorbidities such as obesity, cardiovascular risk factors, and diseases of the gastrointestinal tract were also recorded (Table 1). EFTR was performed on the basis of the following indication: 69.9% (in *n* = 160) adenomas with negative lifting sign (recurrent adenomas in 23.1%, residual adenomas after incomplete resection in 27.9% and treatment naive adenomas in 18.8%), 21.0% (*n* = 48) colorectal adenocarcinoma, and 7.9% (*n* = 18) subepithelial tumor (e.g., NET). Other indications included 2 DALMs and a full-wall biopsy for diagnosis of aganglionosis (Hirschprung's disease). Of note, eFTR for resection of DALMs was performed as an individual decision in patients who were unfit for colectomy due to age and comorbidities. Localization of eFTR was as follows: ascending colon in 25.8%, rectum in 25.3%, sigmoid colon in 14.8%, transverse colon in 11.8%, descending colon in 8.3%, coecum in 8.3%, appendix orifice in 3.9%, and colonic surgical anastomosis (after left-sided hemicolectomy in 2, low anterior resection in 1 and ascendo-descendostomy in 1 patient, respectively) in 1.7%*.* The average diameter of the lesions was 16.32 ± 7.71 mm as measured endoscopically by using an opened forceps or a snare with defined size.

### Technical success rate of eFTR

Overview of results of the whole study collective is shown as a flow chart (Fig. 1). Regarding technical success rate (macroscopically complete), eFTR could be performed in 83.8% (*n* = 192/229, binomial confidence interval: 78.4–88.4%). In 6.6% (*n* = 15/229), the resection of the lesion was completed by using a conventional snare (after technical malfunction of FTRD®-snare, incomplete resection after eFTR and macroscopic evidence of residual tissue near the OTSC). In 3.5% (*n* = 8/229 cases), advancement of the endoscope with the mounted FTRD system to the target lesion was not possible due to diverticular disease, an elongated tortuous colon or presence of adhesions (Table 2). In 12.7% (*n* = 29/229), eFTR was not completely possible. The most frequent causes for technical failure were insufficient grasping of tissue with grasping forceps (*n* = 7) and incomplete retraction of tissue, the application cap with superficial resection (*n* = 8).

**Table 2:** Procedural data of eFTR (entire study population, n = 229) showing technical success rate and details for technical failure (Details of sedation could be assessed in 143 patients)

Procedural data (entire study collective)	
Use of anesthetics (*n* = 143)	
Propofol, mean in mg (range)	454.68 (0–1910)
Midazolam, mean in mg (range)	1.12 (0–10)
Catecholamines required (Arterenol-noradrenaline), *n* (%)	8 (5.6%)
Patient intubated endotracheally, *n* (%)	3 (2.1%)
Technical success, *n* (%) *n* = 229	
Target lesion not reached with FTRD	8 (3.5%)
Advancement of endoscope not possible	− 6 (2.6%)
Dislocation of FTRD clip or cap during advancement	− 2 (0.9%)
Technical failure during resection	29 (12.7%)
Marking of lesion not feasible	1 (0.4%)
Fixation of tissue not possible	7 (3.1%)
Insufficient pull into FTRD-cap, superficial resection	8 (3.5%)
Primary clip malfunction	1 (0.4%)
Avulsion of tissue from grasper after clipping	3 (1.3%)
Macroscopic evidence of residual lesion	9 (3.9%)
Resection technical successful	192 (83.8%)
[95% Binominal confidence interval]	[78.4–88.4%]
Primary	177 (77.3%)
After secondary resection with conventional snare	15 (6.6%)
Histologically confirmed full-thickness resection, *n* (%), *n* = 210 [95% binomial confidence interval]	189 (90.0%) [85.1–93.7%]
R0 Resection, *n* (%), *n* = 154 [95% Binomial Confidence Interval]	119 (77.2%) [69.8–83.6%]
Duration of eFTR, mean in min (range), *n* = 79	54.9 (10–163)
Duration of the inpatient stay, median (days, range), *n* = 177	4.48 (1–32)

### Procedural data

Procedural data including length of hospital stay and details of sedation are summarized in Table 2. An average of 454.68 ± 305.58 mg propofol, 1.12 ± 1.74 mg midazolam, and 6.57 ± 13.74 mg buscopan was administered during the procedures (i.v.-application, details of sedation could be assessed in 143 patients). Peri-interventional administration of catecholamines was necessary in 5.6% (*n* = 8) of cases. In 2.1% (*n* = 3) cases, an endotracheal intubation was performed for the intervention. Duration of eFTR (as defined as entire procedure time: time interval between the insertion of the coloscope and the final endoscopic evaluation after endoscopic full-wall resection using FTRD; obtained in 3 hospitals only: Bogenhausen, Erlangen, and Aalen) ranged from 10 to 163 min (mean 54.9 min). The median duration of the inpatient stay was 4.48 ± 2.93 days (range: 1–32 days). One patient stayed in hospital for 32 days due to a septic urinary tract infection so eFTR was performed after successful recovery from sepsis.

### Histology/R0 resection

All resected specimens were analyzed by the local pathologist regarding histology and R0 status. Of the resectates obtained (*n* = 210), 190 were resected en bloc (90.5%). A histologically proven full-wall excidate (defined as resection specimen including *muscularis propria*) was confirmed in 90.0% (189 of 210 resected lesions, binomial confidence interval 85.1–93.7%) while overall R0 resection rate was achieved in 77.2% (119/154 lesions; binomial confidence interval: 69.8–83.6%). Table 3 gives an overview of histopathological results.

**Table 3:** Overview of histopathological results of total cohort

Histological results (*n* = 210)	
Specimens free of dysplasia	56 (26.0%)
Regular colon wall	− 12 (5.7%)
Fibrosis, inflammation or atrophic colon wall	− 139 (18.6%)
Hyperplasia	− 5 (2.4%)
Adenomas	107 (51.0%)
Low-grade tubular/ tubulovillous	− 59 (28.1%)
High-grade tubular/tubulovillous	− 23 (11.0%)
Sessil serrated	− 23 (11.0%)
Low-grade villous	− 1 (0.5%)
High-grade villous	− 1 (0.5%)
Adenocarcinoma	36 (17.1%)
pT1 L0 V0 Pn0	− 29 (13.8%)
pT1 L1 V0 Pn0	− 3 (1.4%)
pT2 L0 V0 Pn0	− 4 (1.9%)
NET (all pT1 L0 V0 Pn0)	9 (4.3%)
DALM (max. high grade)	2 (1.0%)

Furthermore, the size of the resection specimens was accurately measured by the pathologists. Average size of all specimens was 22.2 mm × 18.4 mm (409,54 mm^2^).

56 patients underwent eFTR because of macroscopically conspicuous lesions, compatible with adenomatous tissue. In the majority, 29/56 biopsies from these lesions were taken before. However, no adenoma or dysplasia could be confirmed by full-thickness histology. Remarkably, the histopathological evaluation of resection specimen showed only fibrosis, hyperplastic tissue, or pseudopolyps. This occurred especially in the relapse situation. We assume that in these 56 patients, lesions had been already removed successfully en bloc (by biopsy prior to eFTR).

107 (51.0%) of all histological results showed adenoma (details of subtypes are shown in Table 3). 104 (97.2%) of these eFTRs were performed for resection of difficult adenoma. Of all eFTRs treating adenocarcinoma (diagnosed prior to eFTR, no R0 resection in initial histology), histological results of the full-thickness resectates confirmed adenocarcinoma in only 18 patients.

Overall, the group of patients with histologically confirmed adenocarcinoma included 36 (17.1%) patients (29 cases of pT1 L0 V0 Pn0, 3 cases of pT1 L1 V0 Pn0, and 4 cases of pT2 L0 V0 Pn0). In 17 (47.2%) of these cases, surgical revision was recommended. Altogether, curative endoscopic resection of histologically confirmed adenocarcinoma (as defined by R0, no surgery mandatory) could only be achieved in 41.7% (15/36). In patients undergoing eFTR for therapy of DALM (in 2 cases), final full-thickness histology confirmed this diagnosis (maximal high grade). In one patient with suspicion of Hirschsprung's disease, the resectate showed no evidence of aganglionosis.

### Subgroup analysis

Subgroup analyses were performed regarding technical success and R0 resection state, considering different indications for eFTR, lesion size, and localization (Table 4).

**Table 4:** Subgroup analysis showing correlation of indication for eFTR, histology, lesion size, localization with technical success, and R0 resection rate (denominators of indication, lesion size, and localization vary in the 2 columns for each line. This can be due to the fact that technical success can be defined for every case in the study collective whereas R0 status can only be determined for dysplastic “risk lesions” which were resected successfully)

Subgroup	Technical success, *n* (%)	R0 Resection, *n* (%)
Indication		
Recurrent adenoma	43/53 (81.1%)	33/39 (84.6%)
Incompletely resected adenoma	57/64 (89.1%)	34/45 (75.0%)
Treatment naive adenoma	34/43 (79.1%)	28/38 (73.7%)
Histological result		
Tissue free of dysplasia	51/56 (91.1%)	− / − ( −)
Adenoma (max. high grade)	95/107 (88.8%)	84/107 (78.5%)
Adenocarcinoma	34/36 (94.0%)	25/36 (69.4%)
NET	9/9 (100%)	9/9 (100%)
Lesion size		
< 10 mm	20/21 (95.2%)	13/14 (92.9%)
10–20 mm	65/75 (86.0%)	40/56 (71.4%)
> 20 mm	11/24 (45.8%)	12/17 (70.6%)
Localization		
Colon	140/167 (83.8%)	92/117 (78.6%)
Proximal colon*	93/114 (81.6%)	70/83 (84.3%)
Distal colon**	47/53 (88.7%)	22/34 (64.7%)
Rectum	49/58 (84.5%)	26/35 (74.3%)
Surgical anastomosis	3/4 (75%)	1/2 (50%)

*Including Coecum, Appendiceal orifice, ascending and transverse colon

**Including descending colon and sigmoid

#### Indication

The subgroup of patients with difficult (non-lifting) adenomas as indication for eFTR included 160 patients with negative lifting sign (recurrent adenomas in 23.1%, residual adenomas after incomplete resection in 27.9%, and treatment naive adenomas in 18.8%, see Table 1). In this subgroup, eFTR could not be performed in 16.3% (26/160) due to technical problems or unsuccessful advancement of the endoscope to the target lesion. Technical success varied from 89.1% in incompletely resected adenoma to 79.1% in treatment naive adenoma and R0 rate from 84.5% in recurrent adenoma to 73.7% in treatment naive adenoma.

#### Histological result

When eFTR was performed for treatment of difficult adenomas, R0 resection was achieved in 78.5% (84/107) compared to 69.4% (25/36) in patients with endoscopic resection of adenocarcinomas. In patients with NET, R0 resection rate was achieved in 100% (9/9; detailed analysis shown in Table 4).

#### Lesion size

R0 resection rate dropped with increasing lesion size. In target lesions < 10 mm, the technical success rate was 95.2% while R0 status could be achieved in 92.9%. In lesions between 10 and 20 mm, the technical success rate was 86.0%, while R0 status was achieved in 71.4%, respectively. In target lesions > 20 mm, the technical success rate decreased to 45.8% while R0 status could be achieved in only 70.6%.

### Procedure-related immediate or late complications after eFTR

Complications associated with eFTR were classified (1) as "moderate" (prolongation of hospital stay, further medical intervention required) or "severe" (potentially life threatening, surgery required) and (2) as procedure-related immediate or late.

Overall, moderate complications after eFTR (within 0–12 h) occurred in 8.3% (*n* = 19) of cases (Table 5). These was arterial bleeding requiring treatment (endoscopic hemostasis, but no blood transfusions) in 3.1%, (*n* = 7), oozing bleeding in 2.6% (*n* = 6) (endoscopic hemostasis required, no blood transfusions), hypotension requiring catecholamines in 2.2% (*n* = 5), as well as edematous narrowing of the intestinal lumen in 0.4% (n = 1).

**Table 5:** Procedure-related complications after eFTR (entire study population, *n* = 229 patients)

Immediate adverse events (0–12 h after intervention)	
Moderate*	19 (8.3%)
Arterial bleeding (hemostasis required)	7 (3.1%)
Diffuse bleeding (hemostasis required)	6 (2.6%)
Relevant narrowing of colonic lumen (clinic surveillance required)	1 (0.4%)
Relevant hypotension (catecholamines required)	5 (2.2%)
Severe**	
Perforation with requirement of secondary defect closure	1 (0.4%)
Secondary adverse events (12–14 days after intervention)	
Moderate*	18 (7.9%)
Fever	2 (0.9%)
Bleeding (repeated endoscopic intervention required)	15 (6.6%)
Postpolypectomy syndrome	1 (0.4%)
Severe**	
Acute appendicitis with requirement of laparoscopic appendectomy	2 (0.9%)
All adverse events	
Moderate	37 (16.2%)
Severe	3 (1.3%)

*Moderate: adverse events requiring medical or repeated endoscopic intervention and/or prolonging hospital admission

**Severe: requiring surgical therapy and/or potentially life threatening

Late complications after eFTR (defined as complications occuring from 12 h—14 days after eFTR) were observed in 7.9% (*n* = 18 patients, see Table 5). Altogether, we observed 2 patients with fever, 15 patients with post-interventional bleeding and one case of post-polypectomy syndrome. All patients were managed successfully with conservative therapy (e.g., intravenous antibiotics, endoscopic hemostasis).

**Fig. 2:**
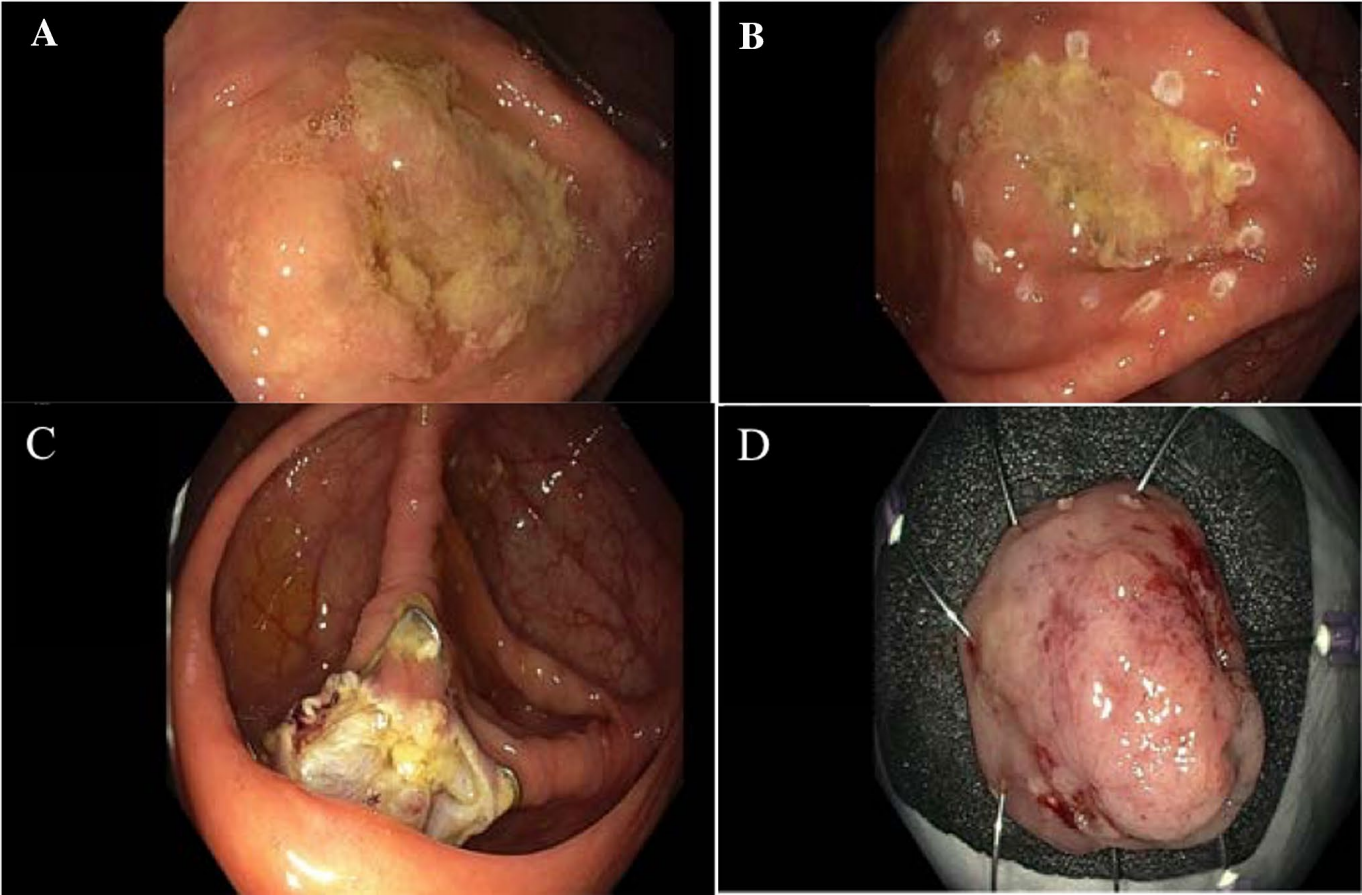
**A** Endoscopic image of the non-lifting adenoma involving the appendiceal orifice (diameter of lesion approximately 20 mm), **B** Lateral markings before full-thickness resection (FTRD), **C** Endoscopic view showing the resection site with FTRD clip securing perforation closure of colonic wall; **D** Resection specimen pinned down on rubber foam before immersion in formalin ( source: Bogenhausen hospital)

Overall, the rate of severe complications was low (1.3%, *n* = 3). Of those, there were 2 cases of acute gangrenous appendicitis requiring operation. In one female patient (age 58 years), eFTR of a flat non-lifting adenoma involving the appendiceal orifice was performed (at Bogenhausen hospital). The diameter of the lesion was 20 mm. For prevention of acute appendicitis, antibiotics were given for 7 days immediately after endoscopy. The procedure was technically successful (Fig. 2A–D) and complete (histology: tubular adenoma with low-grade dysplasia). However, the patient presented 9 days after intervention with right lower abdominal pain. Emergency laparoscopy demonstrated an acute gangrenous appendicitis resulting in a large inflammatory mass necessitating conversion to open surgery. An ileocecal resection was necessary because severe inflammation extending to the cecum precluded an appendectomy or cecal resection (Figs. 4–6, Supplement). Histological examination of the ileocecal specimen revealed extensive transmural necrosis of the appendix and the FTRD clip still securing closure of the colonic wall (Figs. 4–6, supplement). In one case, a severe peri-interventional complication in the form of a perforation occurred. Secondary defect closure was performed by application of an OTSC.

Regarding both major and moderate adverse event rates, there were no significant differences between the participating centers.

In addition, various laboratory parameters were recorded: The hemoglobin value (hb) remained stable between inpatient admission and discharge after eFTR (mean hb level: 13.49 ± 2.41 g/dl at admission; mean hb level at discharge from hospital: 13.44 ± 2.12 g/dl), the CRP was on average 19.76 ± 32.76 mg/l after surgery (24–48 h after eFTR), and the leukocyte count was 8.73 ± 3.49 /nl after surgery (24–48 h after eFTR).

### Follow-up

Endoscopic F/U data could be obtained from 46.7% (*n* = 92) of 197 cases, in which endoscopic controls were recommended, at the time of data analysis. Of those patients, endoscopic control showed no recurrence in 78.3% (*n* = 72). In 15.2% (*n* = 14), recurrent adenoma was detected while in 6.5% (*n* = 6) an adenocarcinoma could be detected. In these patients, surgical therapy was performed. OTSC was still in situ in 34.2%. 4 patients had died in the meantime while the remaining 51.3% (*n* = 101) of patients were lost to F/U due to disabling disease and were not able to come or refused further F/U. 32 patients did not undergo endoscopic F/U because they had already undergone surgical resection or F/U had not been recommended.

Details of surgical/endoscopic revisions performed after eFTR are shown in Table 6. When summarizing all operations performed in our cohort, 12.2% (*n* = 28) patients were referred for (elective) surgery after eFTR due to the following indications: non-R0 high-grade adenoma (2 segmental colectomies in 2 cases), non-R0, or locally advanced adenocarcinoma (4 segmental colectomies and 7 hemicolectomies in 11 cases) and operations performed due to technical failure of eFTR (4 segmental colectomies, 7 hemicolectomies, 1 transanal microsurgery, and 1 other colorectal operation). 6 patients without extracted specimen were treated as follows: in one case, eFTR had to be repeated, in 3 cases, surgery was recommended but rejected by the patients, and in 2 cases, patients had do undergo close follow-up inspections in case of low-grade dysplasia secured by pre-biopsy (surgery in both cases not possible due to advanced patient’s age or comorbidity*).* 2 emergency surgeries (Ileocecal resections) were required treating gangrenous appendicitis. In 3.9% (*n* = 9) cases, surgery was recommended (1 case of high-grade adenoma apart from 3 cases of technical failure of eFTR), but rejected by the patient. In 2.6% (*n* = 6) cases, the eFTR had to be repeated (once in a case of non-R0 resected low-grade adenoma, twice in case of non-R0 resected high-grade adenoma and in 2 cases of adenocarcinoma; in 2 cases, eFTR had to be repeated due to technical failure). Between eFTR and follow-up, an average of 8.73 ± 9.49 months passed (1–48 months).

**Table 6:** Requirement of surgical/endoscopic revision,* n* (%)* n* = 229

Elective surgery performed	28 (12.2%)
Segmental colectomy	10 (4.4%)
Hemicolectomy	14 (6.1%)
TEM	2 (0.9%)
Surgery without precision	2 (0.9%)
Repeated EFTR	6 (2.6%)
Emergency surgery required (Ileocecal resection)	2 (0.9%)
Surgical revision recommended, but not performed or refused by patient	9 (3.9%)
Endoscopic follow-up inspection, *n* (%) *n* = 197	
No proof of endoscopic follow-up inspection	101 (51.3%)
Inspection performed, no recurrence	72 (36.5%)
Inspection performed, recurrence of adenoma	14 (7.1%)
Inspection performed, adenocarcinoma	6 (3.0%)
Patient deceased	4 (2.0%)
Time between intervention and follow-up inspection, mean in month (range) *n* = 89	8.73 (1–48)
No recurrence	− 8.78 (1–48)
Recurrence of adenoma	− 8.79 (1–21)
Adenocarcinoma	− 8.00 (1–26)
OTSC in situ at the time of endoscopic inspection *n* = 76	26 (34.2%)
Symptoms occurring after clinical discharge reported at first follow-up inspection, *n* (%) *n* = 59	
Abdominal pain, soreness	1 (1.7%)
Meteorism	1 (1.7%)
Bacterial bowel infection (antibiosis required)	1 (1.7%)
Mucous stool	1 (1.7%)
Rectal bleeding	1 (1.7%)

### Comparison of different hospitals

Absolute numbers of eFTR-procedures varied between the participating 7 hospitals (minimum 11, maximum 50 cases). For more details view Table 7.

**Table 7:** Overview of different hospitals showing number of procedures and observation period

Center	Bogenhausen	Neuperlach	Dritter orden	Erlangen	Kaufbeuren	Neumarkt Obpf	Aalen	Combined study collective
n	50	33	11	22	48	44	24	232
Observation period	01/16–08/18	01/15–04/18	03/17–07/18	06/15–7/18	11/14–11/18	11/14–12/18	07/15–06/19	11/14–06/19

### Effects of volume and number of endoscopists

**Fig. 3:**
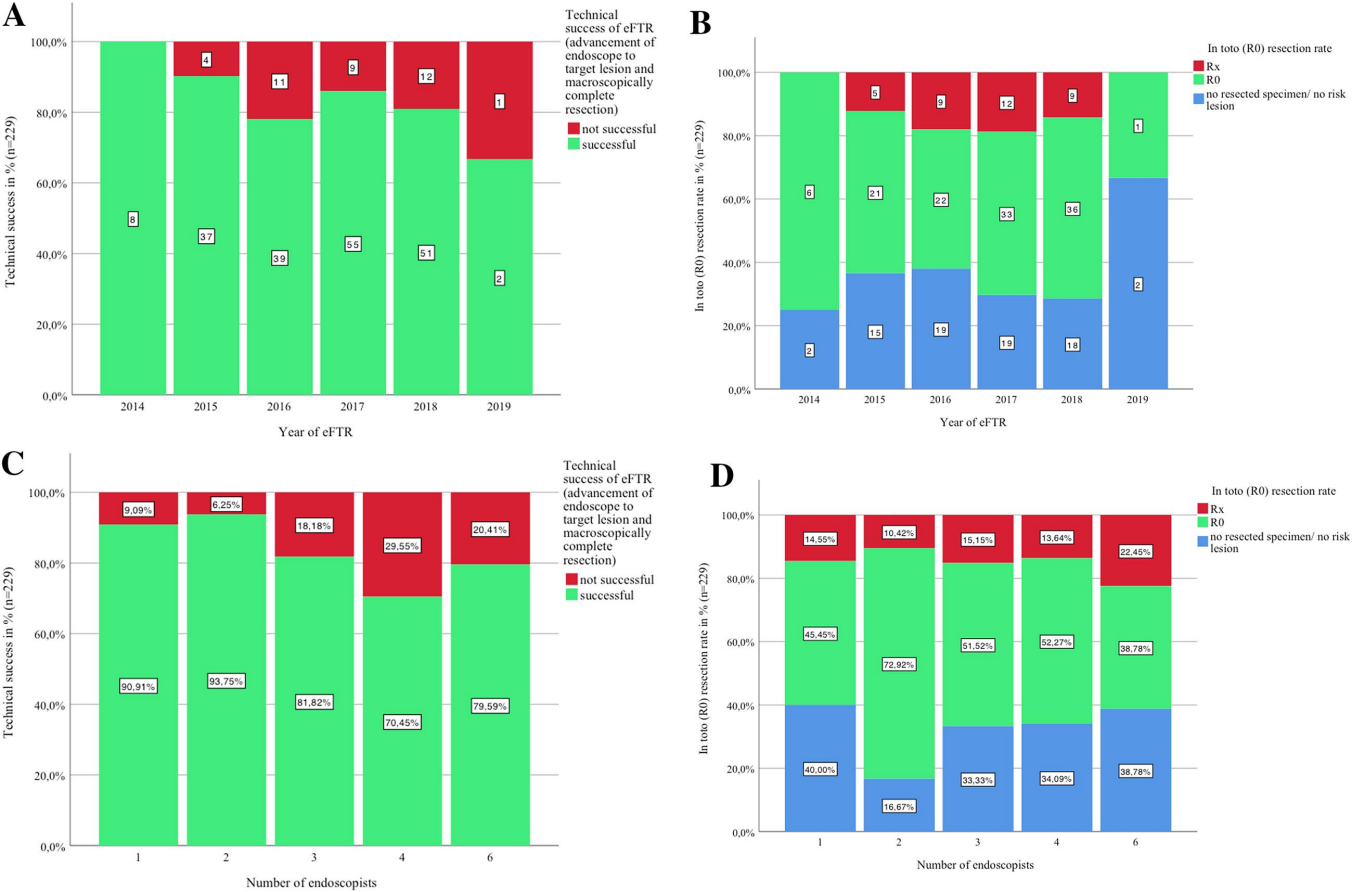
**A**–**D** Correlation of technical success and R0 resection rate with experience (assessed by volume of eFTR over time, **A**, **B**) and number of endoscopists performing FTRD procedures (**C**, **D**). Lesions that required close follow-up or surgery were qualified as "risk lesions"

EFTRs were performed in 7 different hospitals by a total of 22 endoscopists. On average, 1.75 (1 to 5) physicians and 1.85 (0 to 4) assistants were involved in the examinations. Therefore, the different participating hospitals were compared regarding volume of FTRD-endoscopies and number of endoscopists performing eFTR. Technical success and R0 resection rates of eFTR were analyzed for each single year from 2014 to 2019 possibly demonstrating a “learning curve” regarding improved technical and histological resection rates over time, due to greater experience of the endoscopist (Fig. 3A–D). Similar to ‘WALL RESECT,’ we did not observe a clear improvement in resection success over time [20]. However, we could see an effect of the number of endoscopists regarding outcome of eFTR (Fig. 3C, D). Technical success (> 90.9%) and histological R0 resection rates were superior if intervention was performed by one or two endoscopist, compared to 3 or more (< 81.2%).

## Discussion

In this case series, colonoscopic eFTR was performed in 229 patients mainly for treatment of difficult adenomas (69.9%) and gastrointestinal adenocarcinomas (21.0%). Technical success rate of eFTR was achieved in 83.8% while histologically complete resection (R0) was achieved in 77.2% demonstrating histologically proven full-wall excidate in 90.0%. No clear improvement of technical success and R0 resection rate could be observed over time by the performing endoscopists. Procedure-related, mostly moderate complications were observed in 17.5% including 2 cases of acute gangrenous appendicitis requiring operation.

Real-world data of eFTR performed by endoscopists in hospitals with a lower level of care and outside of published studies are rare [21–29]. In particular, it is not clear whether the positive results of the published studies obtained in referral centers can be transferred to the performance quality in other hospitals.

The prospective ‘WALL RESECT’ multicenter study which was published recently demonstrated reasonable technical efficacy of eFTR with the FTRD® system especially in lesions ≤ 2 cm with acceptable complication rates [20]. In this study, the overall primary endpoint (complete en bloc and R0 resection) was reached in 76.9% while EFTR was technically successful in 89.5% [20]. In 90.5%, en bloc resection was obtained. In patients with benign histology (e.g., difficult adenomas), R0 resection rate was even 77.7% [20]. Adverse events were described in 9.9% while 2.2% of patients had to undergo emergency surgery [20]. Three-month follow-up revealed recurrent/residual tumor in 15.3% [20]. Therefore, the authors concluded that eFTR with the FTRD® system demonstrated good overall technical efficacy in benign lesions ≤ 2 cm with acceptable safety while in malignant lesions, it can not be recommended as primary therapy due to the low curative resection rate [20]. However, in this study, eFTR was performed by high-endoscopic technicians with broad experience in innovative and experimental endoscopy which may explain the positive results of this study.

The first US multicenter study on FTRD in the therapy of colonic lesions which were published recently by Ichkhanian et al. showed consistent results with R0 resection rate in 82.7% while technical success was achieved in 84.2% [29]. In this study, the rate of adverse events was also low [29].

In the present case series, a retrospective pooled analysis was obtained evaluating endoscopic full-wall resection procedures using a FTRD® system performed in 7 hospitals in Southern Germany of different care levels. Especially, we wanted to find out whether technical success (as defined by successful advancement of the cap-mounted endoscope to the target lesion and macroscopically complete resection), en bloc (R0) resection rate, and number of procedure-related complications were comparable with the outcomes of ‘WALL RESECT’. Furthermore, we wanted to demonstrate wether there was a possible “learning curve” resulting in an improved rate of technical success and R0 resection over time, due to greater experience of the endoscopist. Therefore, the different participating hospitals were analyzed regarding their volume of FTRD®-endoscopies and the number of endoscopists performing eFTR.

Our patient cohort consisted of 229 patients, which represents one of the largest published cohorts having analyzed eFTR so far. When comparing indication for eFTR and localization of target lesions with the study population of ‘WALL RESECT,’ we found similar percentages between these two cohorts.

Based on the results of these published studies, we defined the targeted success rate (technical feasibility, R0 resection status) for the present case series [20, 29]. Technical success rate and R0 resection state in the present cohort were almost comparable (83.8% and 77.2%, respectively). Additionally, histologically proven full-wall excidate could be obtained even more often (in 90.0%) although the average size of the resected lesions was larger (16.32 mm, range 3–50). Comparable to ‘WALL RESECT,’ we could demonstrate a correlation between lesion size and successful R0 resection ranging from 92.9% (in lesions < 10 mm) to 70.6% (in lesions > 20 mm).

However, advancement of the endoscope with the mounted cap to the target lesion was not possible in all cases and failed in 8 patients (3.5%) due to diverticulosis, an elongated tortuous colon or presence of adhesions. This discrepancy might be due to the special expertise of endoscopists performing eFTR in ‘WALL RESECT’ which was partly involved in the development of the FTRD® system. Although all resections in the present analysis were performed by endoscopists with broad expertise in endoscopy (including EMR and OTSC placement), not everybody had undergone a hands-on training by the manufacturer.

Remarkably, the rates of adverse events (17.5%) were higher in our cohort. However, the vast majority of procedure-related complications was only moderate and severe.

In the study published by Schmidt et al., successful eFTR was performed in 34 difficult-to-resect adenomas at the appendiceal orifice resulting in three cases of acute appendicitis [20]. In two patients, symptoms were mild and conservative treatment was successful while the third patient required laparoscopic appendectomy [20]. Therefore, the authors emphasize in the discussion that closure of the appendiceal orifice with the FTRD® may implicate the risk of acute appendicitis without any complication.

Although eFTR near the appendix orifice was only performed in 3.9% in our cohort, we observed 2 cases of acute gangrenous appendicitis requiring operation. Therefore, one of the participating centers (Bogenhausen hospital) is very cautious about this indication and informs the patient about this special risk. However, the available literature reports a quite acceptable risk of eFTR in the area close to the appendix [31, 32].

Of note, no perforation requiring subsequent surgical revision was reported in our cohort. Furthermore, intervention was performed under conscious sedation with a need of tracheal intubation in only 2.1%. This is in line with previously published literature [20–22, 29].

Another aim of the present study was the question whether there might be a “learning curve effect” demonstrating an improvement of technical success and R0 resection rate over time, due to greater experience of the endoscopist. Since in the present analysis interventions were performed in 7 different hospitals by a total of 22 endoscopists, the participating hospitals were analyzed regarding their volume of FTRD®-endoscopies for each single year from 2014 to 2019 and the number of endoscopists performing eFTR (Fig. 3A–C). Similar to ‘WALL RESECT,’ we did not observe a clear improvement in resection success during the course of time (20, Fig. 3A, B). However, we could see an effect of the number of endoscopists regarding procedure-related outcome (Fig. 3C, D). Rates of technical success (> 90.9%) and histological R0 resection were superior if intervention was performed by one or two endoscopists only, compared to 3 or more (< 81.8%).

The limitations of this case series should be discussed. First, the present study was performed retrospectively. In this study, the interventions were usually performed by the senior physician in charge of endoscopy, in each case by an interventional endoscopist with many years of experience. In this respect, the results are not easily transferable. Especially when it comes to advancing the device to the site of the intervention in the colon, the examiner must have many years of expertise in performing coloscopy. Since the data were collected retrospectively, certain details could not be recorded for methodological reasons (e.g., duration of advancement of the colonoscope with already mounted FTRD).

The average length of stay was 4.48 days, which is rather long for an MIS procedure, compared to EMR, ESD, and TAMIS, which can be done as day surgery. Since the present case series was evaluated retrospectively, some of the patients included were admitted to hospital for various other reasons (anemia clarification, clarification of GI symptoms, etc). Since FTRD is still a new and innovative procedure, all patients were treated for a sufficiently long inpatient stay until the clinical freedom from symptoms could be clearly established after the intervention. These factors explain the sometimes significantly longer inpatient stays.

The implications to research and practice should be addressed. The armamentarium of the interventional endoscopist is expanding rapidly. However, some endoscopic options are only feasible in a very limited number of suitable patients. In everyday endoscopic work, there is a great difference between basic endoscopic care and highly complex interventions, which are performed by renowned experts, for example, in the context of live demonstrations. Since results concerning feasibility of certain endoscopic procedures are usually written by such experts, the question regularly arises whether these results can be transferred to the broad. It would therefore be important to keep a register for certain endoscopic procedures (such as endoscopic full-wall resection) in order to systematically record the quality of results.

The indication for prophylactic antibiotic administration (single shot) was derived in analogy to the common practice of surgical segment resections. Since eFTR using FTRD is an innovative procedure, this procedure was chosen in order to optimize the conditions as much as possible. In our case series, no significant increase of infectious complications after eFTR was observed. The manufacturer (OVESCO, Tübingen, Germany) does not have any binding guidelines in this respect: a procedure by analogy to surgical therapy is recommended. Future systematic studies must show whether prophylactic antibiotic administration is actually necessary or not.

To conclude, the present pooled analysis confirmed the emerging role of eFTR as a feasible, effective, and safe minimally invasive endoscopic technique demonstrating high success rate in the resection of various lesions throughout the gastrointestinal tract with only few severe complications. In selected patients, it represents an alternative to surgical therapy. However, since curative resection of adenocarcinomas was too low, this innovative procedure should be primarily used in benign lesions.

## Electronic supplementary material

Below is the link to the electronic supplementary material.

Electronic supplementary material 1 (PNG 606 kb) Figs. 4-6. (2) Intraoperative view of ileocecal region with acute gangrenous appendicitis precluding an appendectomy resulting in a secondary open ileocecal resection (3) with ileocolonic anastomosis. (4) Ileocecal specimen demonstrating clip still closing the defect of cecal wall
