# Supplementary figures and images for: Technical success, resection status, and procedural complication rate of colonoscopic full-wall resection: a pooled analysis from 7 hospitals of different care levels
Source: Surg Endosc. 2020 Jul 9;35(7):3339–53. doi: 10.1007/s00464-020-07772-5 (PMC8195906; doi:10.1007/s00464-020-07772-5)

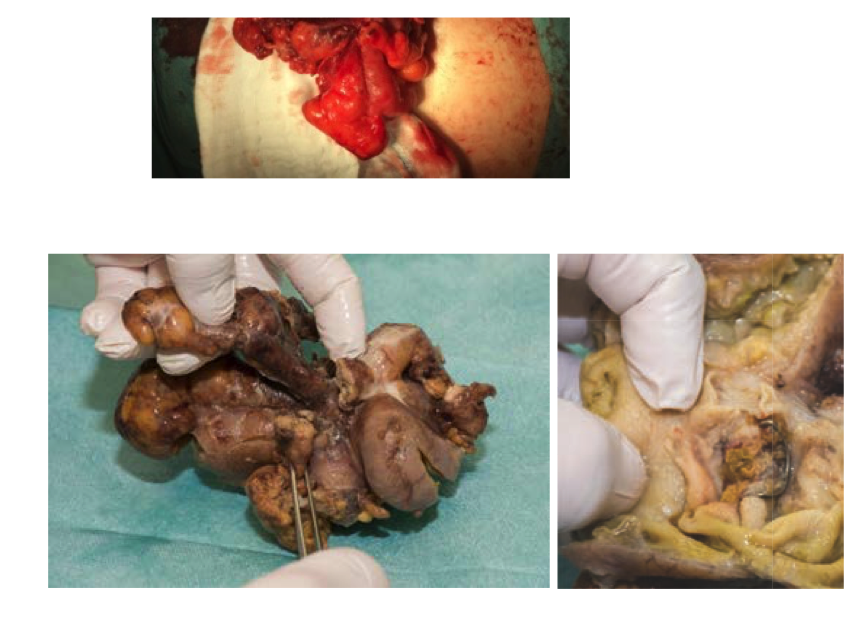

Supplement: Supplementary file 1 — Electronic supplementary material 1 (PNG 606 kb) Figs. 4-6. (2) Intraoperative view of ileocecal region with acute gangrenous appendicitis precluding an appendectomy resulting in a secondary open ileocecal resection (3) with ileocolonic anastomosis. (4) Ileocecal specimen demonstrating clip still closing the defect of cecal wall [file 464_2020_7772_MOESM1_ESM.png]
